# MiR-507 inhibits the migration and invasion of human breastcancer cells through Flt-1 suppression

**DOI:** 10.18632/oncotarget.9163

**Published:** 2016-05-04

**Authors:** Liyan Jia, Wei Liu, Bo Cao, Hongli Li, Chonggao Yin

**Affiliations:** ^1^ Affiliated Hospital, Weifang Medical University, Weifang, 261053, China; ^2^ Medicine Research Center, Weifang Medical University, Weifang, 261053, China; ^3^ College of Nursing, Weifang Medical University, Weifang, 261053, China

**Keywords:** miR-507, Flt-1, molecular mechanism, invasion, PlGF-1

## Abstract

Vascular endothelial growth factor receptor-1/fms-related tyrosine kinase-1 (VEGFR-1/Flt-1) is a tyrosine kinase receptor that binds placental growth factor (PlGF). Flt-1 is also highly expressed in breast-cancer tissues and breast-cancer cell lines. However, the molecular mechanism by which Flt-1 promotes breast-cancer invasion and metastasis by binding to PlGF-1 is unclear. In this study, we discovered that PlGF-1 and Flt-1 played a key role in the migration and invasion of breast cancer. Flt-1 promoted the migration and chemotaxis of breast-cancer cells by binding to PlGF-1. In addition, Flt-1 was confirmed to be a direct target gene of miR-507. miR-507 up-regulation inhibited the invasion and metastasis of breast-cancer cells *in vitro* and *in vivo*. Flt-1 overexpression rescued the invasion partially caused by the ectopic expression of miR-507. miR-507 expression in breast-cancer tissues and cell lines was lower than that in adjacent non-neoplastic tissues and normal cells. Clinical analysis indicated that miR-507 was negatively correlated with tumor differentiation, lymphatic metastasis, and the expression of Flt-1 in breast cancer. Furthermore, we showed that miR-507 down-regulation was due to the hypermethylation of its promotor region. Our results indicated that miR-507 represented potential therapeutic targets in breast cancer by modulating Flt-1.

## INTRODUCTION

Breast cancer is one of the most common malignant tumors among women. More than 100 new cases per 100,000 women aged 55–69 years by 2021 are estimated [1]. The survival rate decreases from 90% for localized breast cancer to 20% for metastatic breast cancer [2]. Cancer metastasis is the toughest problem in cancer therapy. Understanding the molecular mechanisms underlying the invasion and metastasis of breast cancer may provide ways for developing novel antineoplastic therapies.

Vascular endothelial growth factor receptor-1/fms-related tyrosine kinase-1 (VEGFR-1/Flt-1) is a tyrosine kinase receptor that binds placental growth factor (PlGF). The PlGF-1 activates ERK1/2 kinases, which are associated with cellular motility in breast-cancer cells. Some of these activating events are blocked by BP-1, which may explain its anti-tumor activity [3]. FLT1 is highly expressed in breast-cancer tissues and breast-cancer cell lines, but its expression is absent or near background in normal breast tissues [2, 3]. However, the molecular mechanism by which Flt-1 promotes breast-cancer invasion and metastasis by binding to PlGF-1 is also unclear.

MicroRNAs (miRNAs) are small non-coding single-stranded RNAs that posttranscriptionally regulate gene expression by binding partially and complementarily to the 3′-untranslated regions (3′-UTRs) of target mRNA, leading to mRNA inhibition or degradation of translation [4]. Recent studies suggest that miRNAs are involved in cancer metastasis and progression by targeting tumor suppressor genes and oncogenes [5–7]. Let-7a plays an important role as a tumor suppressor gene by targeting HMGA1, which may open novel perspectives for clinical treatments against breast cancer [8]. EGF induces microRNAs that target suppressors of cell migration, whereas miR-15b targets MTSS1 in breast cancer [9]. miR-214 functions as an oncogene in breast cancer, at least partly by promoting cell invasion through the down-regulation of p53 [10]. Until now, the roles of miR-507 in breast cancer have never been described, and this prompted us to identify and validate the role of miR-507 in breast cancer.

In this study, we determined that PlGF-1 and Flt-1 played a key role in migration and invasion of breast cancer. Flt-1 promoted the migration and chemotaxis of breast-cancer cells by binding to PlGF-1. In addition, we found a down-regulation of miR-507 in breast-cancer tissues and cells with respect to the adjacent non-neoplastic tissues and normal cells. Functional assays showed that the expression of miR-507 was inversely correlated with the expression of Flt-1 and the invasive potential of breast cancer. Moreover, Flt-1 was confirmed to be a direct target gene of miR-507.

## RESULTS

### PlGF-1 promoted migration and invasion of breast-cancer cells, and Flt-1 was required for cell migration and invasion mediated by PlGF-1

**Figure 1:**
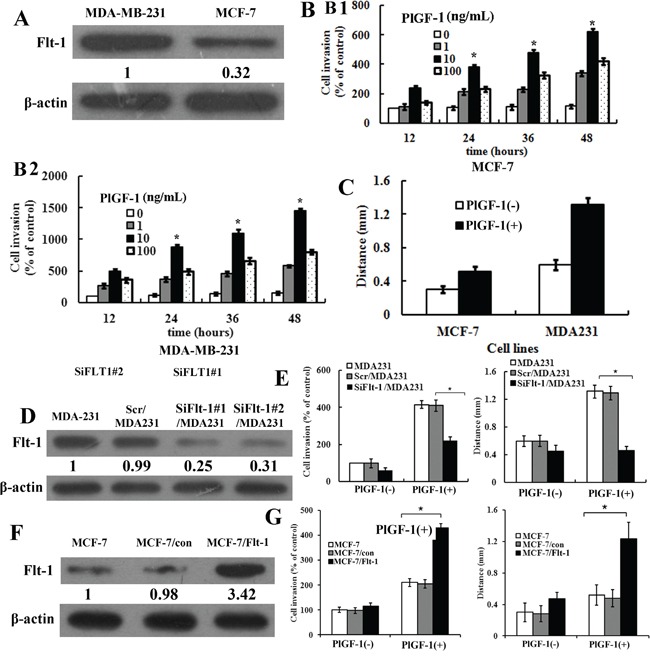
PlGF-1 promoted migration and invasion of breast-cancer cells, and Flt-1 was required for cell migration and invasion mediated by PlGF-1 **A.** Expression of Flt-1 protein in cultured breast cancer cell lines (MCF-7 and MDA-MB-231). β-actin was used as control. Quantification of relative protein levels on three different Western blots is shown below the blots. **B.** B1, Quantitative analysis of cell numbers with various concentrations of rPlGF-1 (0-100 ng/mL) for various time point in MCF-7 cells. B2, Quantitative analysis of cell numbers with various concentrations of rPlGF-1 (0-100 ng/mL) for various time points in MDA-MB-231 cells. Columns, mean of triplicate measurements. Bars, standard deviation. * *P* < 0.05 (two-way ANOVA). **C.** Quantification of wound healing assays in cultured breast-cancer cell lines (MCF-7, MDA-MB-231). The distance of cell migration was measured. rPlGF-1, 10 ng/mL. Columns, mean of triplicate measurements. Bars, standard deviation. * *P* < 0.05 (two-way ANOVA). **D.** Expressions of Flt-1 protein in MDA-MB-231, Scr/MDA231, SiFlt-1#1/MDA231 and SiFlt-1#2/MDA231 cells were detected by Western blot. β-actin was used as control. Quantification of relative protein levels in three different Western blots is shown below the blots. **E.** Left, quantification of PlGF-1-induced penetrated cells were analyzed in Scr/MDA231 and SiFlt-1/MDA231 cells using transwell invasion assay. Columns, mean of triplicate measurements. Bars, standard deviation. * *P* <0.05 (two-way ANOVA). Right, quantification of wound healing assays in Scr/MDA231 and SiFlt-1/MDA231 cells. rPlGF-1, 10 ng/mL. Columns, mean of triplicate measurements. Bars, standard deviation. * *P* < 0.05 (two-way ANOVA). **F.** Expressions of Flt-1 in MCF-7, MCF-7/Con, and MCF-7/Flt-1 cells were detected by Western blot. β-actin was used as control. Quantification of relative protein levels on three different Western blots is shown below the blots. **G.** Left, quantification of PlGF-1-induced penetrated cells were analyzed in MCF-7, MCF-7/con, and MCF-7/Flt-1 cells through transwell invasion assay. Columns, mean of triplicate measurements. Bars, standard deviation. * *P* <0.05 (two-way ANOVA). Right, quantification of scratch assays in MCF-7/con and MCF-7/Flt-1 cells. The distance of cell migration was measured. rPlGF-1, 10 ng/mL. Columns, mean of triplicate measurements. Bars, standard deviation. * *P* < 0.05 (two-way ANOVA).

First, we detected the expression of Flt-1 in different breast-cancer cell lines. Results showed that the breast-cancer cell line MDA-MB-231 had high levels of Flt-1, whereas MCF-7 cell expressed low levels (Figure 1A). To identify the effects of PlGF-1 on breast-cancer cell migration and invasion, cells were starved in a medium without FBS, and then treated with recombination PlGF-1 (rPlGF-1) protein at increased concentrations for different durations. Transwell invasion system consisted of fluid-filled stacked compartments separated by a porous membrane filter coated with Matrigel. Quantitative analysis of cell numbers revealed that the MCF-7 and MDA-MB-231 cell lines had the highest invasion rate that responded to 10 ng/mL rPlGF-1 (Figure 1B). When a scratch was created in the monolayer cells, the distance of breast-cancer cells migration was longer after 24 h with 10 ng/mL rPlGF-1 stimulation than cells without 10 ng/mL rPlGF-1 stimulation (Figure 1C). Subsequently, we used MCF-7 and MDA-MB-231 to investigate the molecular mechanism *in vitro*.

Meanwhile, we detected migration of MDA-MB-231 and MCF-7 cells with or without PlGF-1 stimulation through the wound healing assay. The results showed that the distance of MDA-MB-231 cells migration was longer than the MCF-7 cells with PlGF-1 stimulation. To determine whether Flt-1 played a role in the PlGF-1-induced migration of MDA-MB-231 cells, we inhibited Flt-1 expression in MDA-MB-231 cells through siRNA technology. Stable cell lines of down-regulated Flt-1 expression were selected by puromycin. Transfected cells with a scrambled sequence were designated as Scr/MDA231 cells as a control (Figure 1D). We chose to present the results from SiFlt-1#1/MDA231 designated as SiFlt-1/MDA231 cells as the representative. To determine whether Flt-1 affected the migration and invasion of breast-cancer cells by binding to PlGF-1, we performed wound healing and Transwell invasion assays. The SiFlt-1/MDA231 cells that invaded the Matrigel after 24 h with 10 ng/mL rPlGF-1 stimulation were considerably fewer than the Scr/MDA231 cells. Quantitative analysis of the cell numbers revealed that SiFlt-1/MDA231 cells had a twofold lower invasion rate than Scr/MDA231 cells that responded to 10 ng/mL rPlGF-1 (Figure 1E, left). When a scratch was created in the monolayer cells, the distance of SiFlt-1/MDA231 cells migration was shorter than the Scr/MDA231 cells with PlGF-1 stimulation (Figure 1E, right).

At the same time, stably transfected Flt-1 cell clones were generated through the transfection with pcDNA3.1-Flt-1 plasmid and subsequent selection. All stable clones had similar phenotypes. We chose to present the results from clone 2 (designated as MCF-7/Flt-1 cells) as the representative. MCF-7 cells were also transfected with a pcDNA3.1 vector to establish vector control cells, which were designated as MCF-7/Con. The expression of Flt-1 are illustrated in Figure 1F through Western blot analysis. We also conducted wound healing and Transwell invasion assays in MCF-7 cells. Results showed that MCF-7/Flt-1 cells invading through Matrigel after 24 h with 10 ng/mL rPlGF-1 stimulation were considerably more than MCF-7/Con cells. Quantitative analysis of cell numbers revealed that MCF-7/Flt-1 cells had a twofold higher invasion rate than MCF-7/Con cells that responded to 10 ng/mL rPlGF-1 (Figure 1G, left). When a scratch was created in the monolayer cells, the distance of the MCF-7/Flt-1 cells migration was longer than MCF-7/Con cells with PlGF-1 stimulation (Figure 1G, right). Results indicated that Flt-1 was the receptor of PlGF-1 on breast-cancer cells.

### MiR-507 directly targets Flt-1

**Figure 2:**
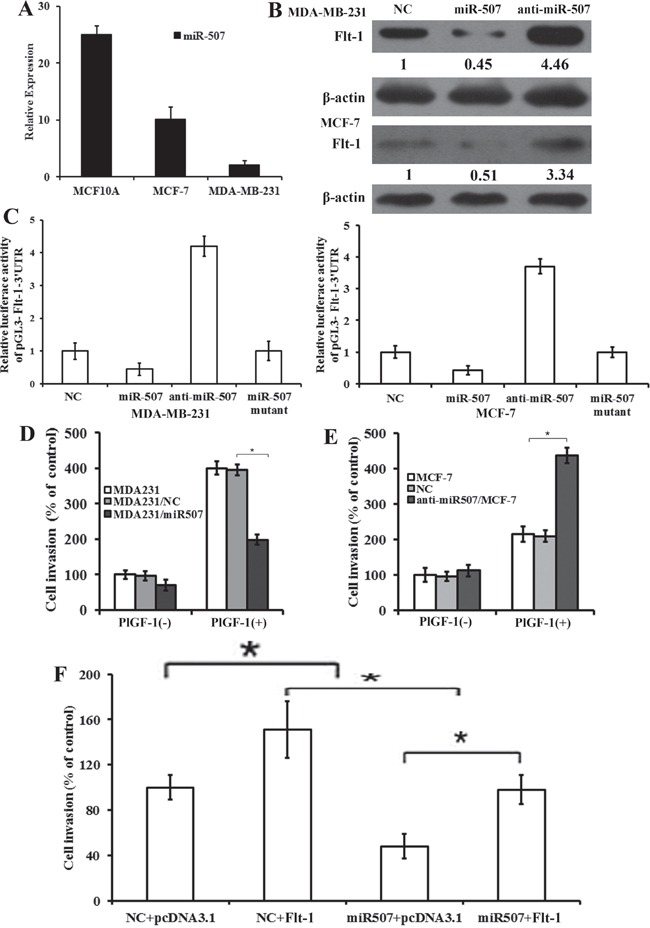
MiR-507 directly targets Flt-1, and Overexpression of miR-507 in breast-cancer cells inhibits invasion **A.** Expression of miR-507 in breast-cancer cell lines through qRT-PCR analysis. Columns, mean of triplicate measurements. Bars, standard deviation. **B.** Western blot analysis of Flt-1 expression in MDA-MB-231 and MCF-7 cells transfected with miR-507 and miR-507 inhibitor. β-actin was used as control. Quantification of relative protein levels in three different Western blots is shown below the blots. **C.** Luciferase activity of pGL3-Flt-1-3′UTR reporter in indicated cells co-transfected with oligonucleotides. **D.** Quantification of PlGF-1-induced penetrated cells were analyzed in MDA-MB-231, MDA231/NC, and MDA231/miR507 cells through transwell invasion assay. rPlGF-1, 10 ng/mL. Columns, mean of triplicate measurements. Bars, standard deviation. * *P* <0.05 (two-way ANOVA). **E.** Quantification of PlGF-1-induced penetrated cells was analyzed in MCF-7, NC, and anti-miR507/MCF-7 cells through transwell invasion assay. rPlGF-1, 10 ng/mL. Columns, mean of triplicate measurements. Bars, standard deviation. * *P* <0.05 (two-way ANOVA). **F.** Quantification of PlGF-1-induced penetrated cells were analyzed in indicated cells through transwell invasion assay. rPlGF-1, 10 ng/mL. Columns, mean of triplicate measurements. Bars, standard deviation. * *P* <0.05 (two-way ANOVA).

We detected the expression of miR-507 in breast-cancer cell lines using qRT-PCR. The results showed that miR-507 was ubiquitously expressed at lower levels in human breast-cancer cell lines than in MCF-10A cell lines (Figure 2A). To conform if Flt-1 expression could be regulated by miR-507, we analyzed the expression of Flt-1 protein through the Western blot 48 h after being transfected by miR-507 mimic (miR-507), miR-507 inhibitor (ant-miR-507) in the MDA-MB-231, and MCF-7 cell lines, respectively. The results showed that Flt-1 expression decreased significantly in miR-507-transfected cells, but increased in ant-miR-507 transfected cells compared with the control cells (Figure 2B). To examine whether miR-507 targets Flt-1 mRNA though its predicted pairing sites, we cloned the 3′-UTR of Flt-1 containing miR-507 targets into a luciferase construct. The results showed that miR-507 regulated Flt-1 expression through a significant reduction or addition of luciferase activity in cells transfected with miR-507 mimic or miR-507 inhibitor, compared with control cells (NC) (Figure 2C). Mutation of miR-507 target sites of Flt-1 made the luciferase activity restore control cells (NC) level (Figure 2C), confirming that 3′-UTR of Flt-1 is a direct target of miR-507. These results suggest that 3′-UTR of Flt-1 is a direct target of miR-507, and miR-507 can directly regulate Flt-1 expression in breast-cancer cells.

### Overexpression of miR-507 in breast-cancer cells inhibits invasion

To verify whether miR-507 plays an important role in the migration and invasion through its target gene, Flt-1, we introduced the synthesized miR-507 mimics into MDA-MB-231 cells and miR-507 inhibitors into MCF-7. Stable miR-507 over-expression in MDA-MB-231 cells (MDA231/miR-507) and stable miR-507 down-regulation in MCF-7 cells (anti-miR507/MCF-7) were obtained. We assessed cell invasiveness through transwell matrigel penetration assay. Overexpression of miR-507 inhibited the invasion of MDA-MB-231, consistent with the effect of silencing Flt-1 (Figure 2D). Down-regulation of miR-507 promoted the invasion of MCF-7, consistent with the effect of up-regulating Flt-1 (Figure 2E). To investigate the contribution of miR-507 to invasion, we ectopically expressed miR-507 together with Flt-1 in MDA-MB-231 cells to evaluate whether Flt-1 may overcome the suppressing effect of miR-507 on cell invasion. MDA-MB-231 cells were co-transfected with NC or miR-507 together with pcDNA3.1 or pcDNA3.1-Flt-1 for 48 hours with the stimulation of PlGF-1. Flt-1 overexpression rescued the invasion partially caused by the ectopic expression of miR-507 in MDA-MB-231 (Figure 2F). Overall, the above results suggest that Flt-1 is a functional target of miR-507, contributing to invasion in MDA-MB-231 cells.

### miR-507 inhibited PlGF-1-induced chemotaxis and actin polymerization of breast-cancer cells

**Figure 3:**
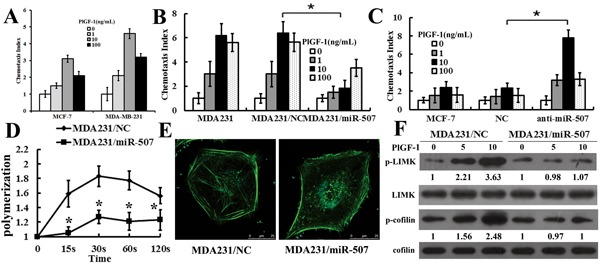
MiR-507 inhibited PlGF-1-induced chemotaxis and actin polymerization of breast-cancer cells **A.** Comparison of PlGF-1-induced chemotaxis of various breast-cancer cell lines (MCF-7 and MDA-MB-231). Columns, mean of triplicate measurements. Bars, standard deviation. **B.** Comparison of chemotactic responses with rPlGF-1 stimulation in MDA-MB-231, MDA231/NC, and MDA231/miR-507. Columns, mean of triplicate measurements. Bars, standard deviation. * *P* < 0.05 (two-way ANOVA). **C.** Comparison of chemotactic responses with rPlGF-1 stimulation in MCF-7, NC/MCF-7, and anti-miR-507/MCF-7. The data were collected in this set of figures from a representative of at least three independent experiments. Columns, mean of triplicate measurements. Bars, standard deviation. * *P* < 0.05 (two-way ANOVA). **D.** Time course of relative F-actin content in MDA231/NC cells and MDA231/miR-507 cells with rPlGF-1 stimulation. rPlGF-1, 10 ng/mL. Bars, standard deviation. * *P* < 0.05 (two-way ANOVA). **E.** Cytoskeleton rearrangements in the MDA231/NC and the MDA231/miR-507 cells were shown by fluorescence assay. Figures showed representative images from three repeated experiments (630×). **F.** Western blot analysis of the phosphorylation of cofilin and LIMK in total cell lysates from MDA231/NC and MDA231/miR-507 cells with 10 ng/mL PlGF-1 stimulation for 0 min, 5 min, and 10 min. Cofilin and LIMK were used as a loading control. Quantification of relative protein levels on three different Western blots is shown below the blots.

To determine whether Flt-1 binding to PlGF-1 affected cell chemotaxis, we performed cell chemotaxis assay. A 24-well chemotaxis model was used to perform PlGF-1-induced cell chemotaxis. PlGF-1 induced the robust chemotaxis in different breast-cancer cell lines, which followed a typical bell-shaped response curve (Figure 3A). Chemotaxis assay showed similar results between parental MDA-MB-231 and MDA231/NC cells. The MDA231/miR-507 cells showed decreased chemotaxis compared with MDA231/NC cells (Figure 3B). The anti-miR507/MCF-7 cells showed increased chemotaxis compared with NC/MCF-7 cells (Figure 3C). These chemotaxis results indicated that Flt-1 played an important role in the PlGF-1-induced chemotaxis of breast-cancer cells.

An F-actin polymerization assay was done to verify that miR-507 over-expression influenced breast-cancer cell migration by reducing F-actin polymerization. The quantitative F-actin polymerization assay revealed that PlGF-1 caused a transient actin polymerization at 20 s and 60 s in MDA231/NC cells, whereas F-actin polymerization that was significantly reduced responded to PlGF-1 stimulation in MDA231/miR-507 cells, which suggested that miR-507 largely restrains the cytoskeleton rearrangement (Figure 3D). The immunofluorescent staining of the F-actins showed that the PlGF-1 induced an increase in the F-actin contents of the MDA231/NC cells, but not of the MDA231/miR-507 cells (Figure 3E). LIM kinase 1 (LIMK) is involved in the regulation of F-actin polymerization [11, 12]. F-actin dynamics is also regulated by the phosphorylating cofilin at Ser3, which is critical for cell chemotaxis and migration [13, 14]. Analysis of PlGF-1-induced phosphorylation of LIMK and cofilin in MDA231/miR-507 cells showed a marked inhibit activation of both cofilin and LIMK, compared with MDA231/NC cells, whereas both total LIMK and cofilin levels remained unchanged (Figure 3F). Overall, all results indicate that miR-507 plays an important role in the PlGF-1 induced LIMK and cofilin recycling.

### Flt-1 promoted lung colonization of human breast cancer with PlGF-1 stimulation, and miR-507 inhibited lung colonization of human breast cancer *in vivo*

**Figure 4:**
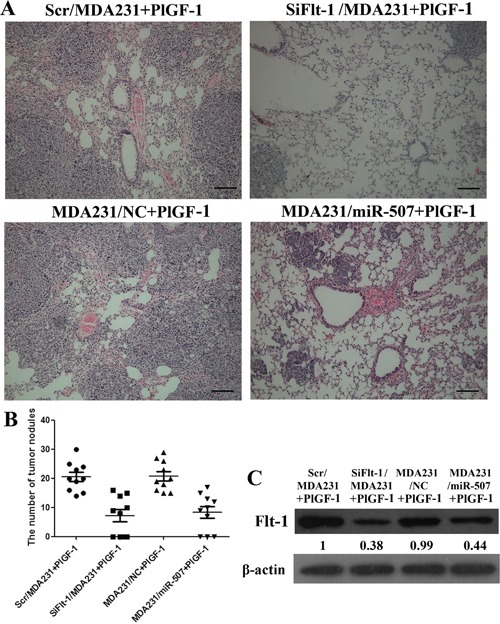
Flt-1 promoted lung colonization of human breast cancer with PlGF-1 stimulation, and miR-507 inhibited lung colonization of human breast cancer *in vivo* **A.** Human tumor foci in mouse lungs were visualized by H&E staining. Scale bar: 50 μm. **B.** Lung metastatic nodules were counted and plotted (*n* = 10). **C.** Western blot analysis for Flt-1 in slices of sectioned implanted tumors; the mice were injected with Scr/MDA231, SiFlt-1/MDA231, MDA231/NC, and MDA231/miR-507 cells. Quantification of relative protein levels on three different Western blots is shown below the blots.

The metastatic properties of breast-cancer cells were analyzed *in vivo* through a xenograft transplant model in SCID mice. We injected Scr/MDA231, SiFlt-1/MDA231, MDA231/NC, and MDA231/miR-507 cells into the mammary fat pads of SCID mouse. When the xenografts were palpable (around 0.5 cm in diameter), intratumor injection of PlGF-1 at 10 ng/kg was performed biweekly for four consecutive weeks. We used H&E staining to examine tumor cell colonies in mouse lungs. The number of metastatic tumor nodules increased in the lungs of mice injected with Scr/MDA231 and PlGF-1 or MDA231/NC compared with that in the lungs of mice injected with SiFlt-1/MDA231 and PlGF-1 or MDA231/miR-507 (Figures 4A, 4B). Simultaneously, the expression of Flt-1 protein in tumor xenograft was down-regulated in mice injected with MDA231/miR507 cells (Figure 4C). The results were consistent with the *in vitro* findings and indicated that Flt-1 induced the invasion of breast cancer by binding to PlGF-1, and miR-507 inhibited the invasion of breast cancer.

### The expression of miR-507 and Flt-1 in breast-cancer tissues

**Figure 5:**
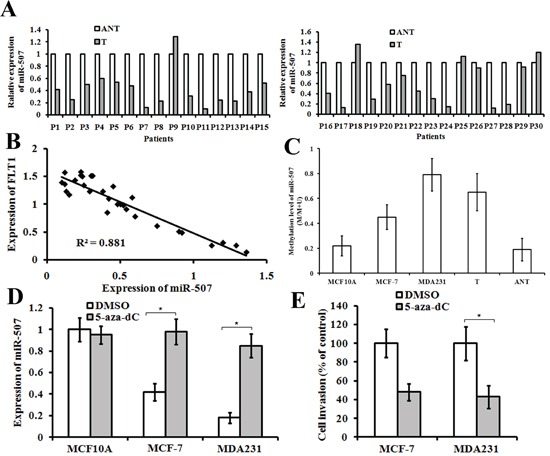
The expression of miR-507 and Flt-1 in breast-cancer tissues, and the function of miR-507 **A.** expression of miR-507 in 30 pairs of frozen invasive ductal carcinoma tissues (T) compared with their corresponding adjacent non-cancerous tissues (ANT). **B.** In human breast-cancer tissues, miR-507 has a negative correlation with Flt-1 protein expression. **C.** Methylation level of the miR-507 genomic region in MCF-10A, breast-cancer cell lines (MCF-7 and MDA-MB-231), breast-cancer tissues (T), and their corresponding adjacent non-cancerous tissues (ANT). **D.** qRT-PCR analysis of miR-507 expression in indicated cells treated with 5 μM 5-aza-dC or DMSO for 72 h. Columns, mean of triplicate measurements. Bars, standard deviation. *P < 0.05. **E.** Comparison of invasive abilities of MCF-7 and MDA-MB-231 cells after being treated with 5 μM 5-aza-dC or DMSO for 72 h. Columns, mean of triplicate measurements. Bars, standard deviation. *P < 0.05.

To further examine the role of miR-507 on human primary tumors, we studied miR-507 expression in 90 archived paraffin-embedded specimens of invasive ductal carcinoma and 30 selected frozen (liquid nitrogen) invasive ductal carcinoma tissues and adjacent non-tumor (ANT) tissues using qRT-PCR. We found that the miR-507 expression level was lower in invasive ductal carcinoma tissues than that in ANT tissues (Figure 5A). Further analysis showed that the down-regulation of miR-507 in breast cancer was associated with tumor differentiation, lymphatic metastasis, and distant metastasis (Table 1), but it did not correlate with age, tumor size, or hormonal status (Table 1). Lastly, we investigated whether miR-507 level was associated with the protein levels of Flt-1 in these patients. The result showed that tumors with a low level of miR-507 tended to express high levels of Flt-1, whereas tumors with a high level of miR-507 tended to express low levels of Flt-1 (Figure 5B). Hence, miR-507 appeared to negatively regulate tumor invasion and metastasis in breast-cancer patients by targeting Flt-1.

**Table 1:** Correlation between clinical features and miR-507 expression in invasive ductal carcinoma patients

Variables	miR-507 expression		*p* Value
	High expression	Low or none expression	
Age (years)			
≤50	20	32	0.456
≥51	28	40	
Tumor size (cm)			
≤5cm	18	34	0.194
>5cm	30	38	
Tumor differentiation			
I	8	12	0.013
II	14	39	
III	26	21	
Lymph node metastasis			
Yes	12	34	0.011
No	36	38	
Distant metastasis			
Yes	13	33	0.029
No	35	39	
Estrogen receptor			
Positive	23	35	0.545
Negative	25	37	
Progesterone receptor			
Positive	18	24	0.391
Negative	30	48	
c-erbB-2			
Positive	21	28	0.366
Negative	27	44	

### miR-507 downregulation was due to hypermethylation of the promoter region of the miR-507 gene

Some tumor suppressor genes, such as miRNAs, can be down-regulated by promoter hypermethylation in human cancers [15, 16]. To elucidate the mechanism of miR-507 down-regulation, we performed BSP with genomic DNA to analyze the methylation level of miR-507. The results showed that the methylation levels of miR-507 were significantly higher in both breast-cancer cell lines and tissues than in immortalized human breast epithelial cell lines (MCF-10A) and ANT. In addition, the methylation levels of miR-507 in MCF-7 cells (low metastasis cell line) were lower than those in MDA-MB-231 cells (high metastasis cell line) (Figure 5C). These findings imply that miR-507 down-regulation is due to hypermethylation of promoter. To find more evidence, we treated MCF-10A, MCF-7, and MDA-MB-231 cells with 5 μM 5-aza-dC for 72 h and found that miR-507 expression was significantly increased in MCF-7 and MDA-MB-231 cells (Figure 5D). Moreover, Matrigel invasion assay showed that 5-aza-dC inhibited the invasive ability of MCF-7 and MDA-MB-231 cells (Figure 5E). Taken together, these findings strongly suggest that miR-507 down-regulation was due to hypermethylation in the promoter region of the miR-507 gene.

## DISCUSSION

Previous studies have shown that PlGF-1-mediated FLT1 activation promoted migration and invasion in MCF-7 (luminal) cells and led to morphologic and molecular changes of epithelial-mesenchymal transition [17]. A number of studies demonstrated that multiple miRNAs can coordinate to inhibit the expression of a target gene by directly binding to its 3′-UTR. Our results showed that Flt-1 binding to PlGF-1 played an important role in breast-cancer cell migration and chemotaxis that is modulated by miR-507. First, we found that PlGF-1 and Flt-1 promoted the invasion and metastasis of breast-cancer cells. Second, miR-507 was sufficiently strong enough to inhibit Flt-1 via direct binding to the Flt-1 3′-UTR. miR-507 inhibited PlGF-1-induced invasion, chemotaxis, and actin polymerization of breast-cancer cells. Flt-1 promoted lung colonization of human breast cancer with PlGF-1 stimulation, and miR-507 inhibited lung colonization of human breast cancer in animal experiments. Finally, Flt-1 is inversely correlated with miR-507. miR-507 modulates Flt-1 expression and is in turn regulated through promoter methylation.

A previous study reported that the administration of miR-507 was effective in inhibiting the growth of tumors formed from A549 cells in nude mice by targeting NF-E2–related factor 2 (NRF2) as well as ME1, a known transcriptional target of NRF2 [18]. By transfecting miR-507 into MDA-MB-231 and MCF-7 cells, we demonstrated that miR-507 could significantly repress Flt-1 protein expression and affect breast-cancer invasion. *In vitro* luciferase assay confirmed that miR-507 exerted its effects by targeting Flt-1. We also observed that miR-507 was ubiquitously expressed at lower levels in human breast-cancer cell lines than in MCF-10A cell lines. In addition, the inverse correlation between miR-507 and Flt-1 expression is evidenced in our clinical analysis. These data are consistent with most of the previous research, further suggesting that miR-507 may perform a tumor-suppressive function. Considering the role of Flt-1 in breast cancer, our results suggested that miR-507 could suppress breast-cancer invasion by directly targeting the 3′-UTRs of the Flt-1 genes.

The ligand-induced cytoskeleton rearrangement is the key to chemotaxis [19]. F-actin polymerization correlates with cellular chemotactic capacity during migration. This remodeling of the actin cytoskeleton is important for the motility and chemotaxis of cancer cells because it consequently influences the metastatic capability of these cells. Our results showed that miR-507 participated in PlGF-1-induced F-actin polymerization to mediate cytoskeletal rearrangement by inhibiting phosphorylation of LIMK and cofilin, which is essential for cell migration [20, 21]. Our results also showed that miR-507 inhibited the PlGF-1-induced actin polymerization by mediating Flt-1. Taken together, our results suggested that miR-507 functioned upstream of LIMK/cofilin and directly regulated PlGF-1-induced actin polymerization.

A more than 50% reduction in expression in primary esophageal squamous cell carcinoma (ESCC) tissue was compared with the corresponding noncancerous tissue and was observed in nine cases (30.0%) for miR-507 [18]. In the current study, we reported that the expression of miR-507 was significantly down-regulated in invasive ductal carcinoma tissues and is inversely correlated with the tumor differentiation, lymphatic metastasis, and distant metastasis. Both our *in vitro* and *in vivo* results support that miR-507 significantly inhibits the invasion and metastasis of invasive ductal carcinoma. These findings demonstrate that miR-507 may function as a tumor suppressor gene in invasive ductal carcinoma.

Carcinogenesis as well as cancer progression result from genetic and epigenetic changes of the genome that leads to dysregulation of transcriptional activity of genes. Promoter hypermethylation of tumour suppressor genes is a kind of epigenetic mechanisms in cancer cells [22]. Epigenetic modifications have been shown to be crucial mediators underlying in miRNA down-regulation and to display a tight correlation with carcinogenesis [16, 23]. Our data demonstrated that the hypermethylation of the upstream promoter of miR-507 led to the down-regulation of miR-507 in breast-cancer tissues and cell lines. Moreover, 5-aza-dC (DNA methyltransferase inhibitor) can increase miR-507 expression in breast-cancer cell lines and can reduce the invasive ability of breast-cancer cells. Based on these findings, the methylation status of miR-507 probably acts as a potential biomarker for breast-cancer prognosis.

In summary, we showed that Flt-1 promoted the migration and chemotexis of breast-cancer cells by binding to PlGF-1. More remarkably, our findings suggest a novel role for miR-507 in inhibiting Flt-1 expression and in suppressing the migration and invasion of breast cancer by inhibiting PlGF-1-induced actin polymerization. Furthermore, low miR-507 expression in breast cancer depends on the hypermethylation of its DNA promoter. Thus, PlGF-1, Flt-1 and miR-507 may be useful prognostic markers for glioma and novel therapeutic targets for breast cancer.

## MATERIALS AND METHODS

### Cell lines

All human breast cancer cell lines were obtained from American Type Culture Collection (ATCC), were regularly inspected for mycoplasma and have been authenticated with a short-tandem repeat profile according to LGC Standards. MCF-10A cells were maintained in Mammary Epithelial Cell Growth Medium (MEGM™). MCF-7 cell line was maintained in Minimum Essential Medium (MEM) containing 10% fetal bovine serum (FBS), and MDA-MB-231 cell line was maintained in L15 supplemented with 10% FBS, at 37°C in a humidified atmosphere of 100% air. All cells used in our experiments were at passages 3 to 15 after obtaining them from the suppliers. Cells were cultured in the absence of serum overnight prior to the treatment with 10 ng/mL PlGF-1 for the indicated periods.

### Cell transfections

MDA-MB-231 Cells were plated in a 35 mm dish for 24 h before transfection in complete medium. Transfection was performed with Lipofectamine 2000 according to the manufacturer's instructions. Stealth siRNAs against human Flt-1 (5′-GAAACCACAGCAGGAAGACGGTCCTATCG-3′ and 5′-TGAAGCGGTTCACCTGGACTGAGACCAAG-3′) and a scrambled siRNA were synthesised by Genechem. Stably transfected cells were obtained by using culture medium with 600 ng/mL puromycin. Single cell clones were isolated for clone expansion. Stable transfected cell clones were maintained in culture medium with puromycin (300 ng/mL). The stable transfected cells are named Si/MDA231 and Scr/MDA231 cells for subsequent studies.

Flt-1 cDNA was cloned into of pcDNA3.1 and was confirmed by DNA sequencing. MCF-7 cells were transfected with pcDNA3.1-Flt-1 plasmid or pcDNA3.1 vector using Lipofectamine 2000 according to the protocol. After 24 h transfection, cells were reseeded into 10 cm culture dishes. Stable transfected cells were obtained by using culture medium with 600 μg/mL G418. Single cell clones were isolated for clone expansion. Stable transfected cell clones were maintained in culture medium with G418 (300 μg/mL). 3′-UTRs of Flt-1 were amplified and then cloned into the downstream of the luciferase gene in a modified pGL3 control vector.

miR-507 mimics, miR-507 inhibitor and negative control mimics (NC) were synthesized by GenePharma company (Shanghai, China). A DNA fragment containing the hsa-miR-507 precursor with 300 bp flanking sequence of each side was amplified into retroviral transfer plasmid pMSCV-puro (GenePharma Company, Shanghai, China). Following transduction, puromycin (600 ng/mL) was used as a selection antibiotic to select the infected cells for 10 days.

### Target prediction of miRNAs

MiRNA.org (http://www.microrna.org/microrna/) and TargetScan 6.0 (http://www.targetscan.org/) were used to search for predicted targets of miRNAs.

### Real-time quantitative reverse transcription-polymerase chain reaction (qRT-PCR)

Total RNA was extracted from breast tissues and cells using TRIzol. The amount of mature miR-507 was analyzed using miR-507 TaqMan MicroRNA Assay kit. The amount of U6 was assessed as the internal standardization. All reactions were carried out in triplicate, and the 2^−ΔΔCt^ method (ΔCT = CT_miR-507_ - CT_U6_) was used to quantify the relative amount of miR-507. Expression of miR-507 in lower metastasis cell lines MCF-7 was set as evaluation criteria, high expression of miR-507 meant that the expression was higher than the criteria level, low expression of miR-507 meant that the expression was lower than the standard or equal the criteria level.

### Luciferase reporter assay

Seed sequences of miR-507 and pairing 3′-UTR sequences of Flt-1 were predicted by TargetScan 6.0 and miRNA.org. Flt-1 3′-UTR luciferase reporter gene plasmid was constructed by inserting the human Flt-1 3′-UTR sequence into the pGL3 vector. The pGL3 construct and the miR-507 mimic, control mimic or miR-507 mutant were co-transfected into cells cultured using Lipofectamine 2000. Luciferase activities were measured using a Dual Luciferase Reportor Assay System in cells transfected after 24 hours.

### Western blot assay

For Western blot, cells were lysed with RIPA buffer supplemented with Protease Inhibitor Cocktail (Roche) and subsequently clarified by centrifugation. Protein concentration was quantified with the BCA Protein Assay Kit (Pierce). The following antibodies were used: Flt-1 (Santa Cruz biotechnology, 1:1000), p-LIMK (Cell Signaling Technology, 1:1000), LIMK (Cell Signaling Technology, 1:1000), p-cofilin (Cell Signaling Technology, 1:1000), cofilin (Cell Signaling Technology, 1:1000), β-actin (Santa Cruz biotechnology, 1:1000) and HRP-linked anti-rabbit IgG (Santa Cruz biotechnology, 1:1000) antibody. All experiments were repeated at least 3 times.

### Chemotaxis assay

Chemotaxis assays were performed by using transwell chambers as described previously [24]. Briefly, PlGF-1 was added into the lower chambers. A polycarbonate filter membrane was placed between the lower and upper chambers. The cells were suspended in binding medium at the density of 1×10^6^ cells/ml and were placed into the upper chambers. The chambers were incubated in a 37°C humidified incubator for 3 h. The membranes were fixed and stained. The number of migrating cells was counted by light microscopy at 400× (high-powered field) in three random fields.

### Transwell invasion assays and wound healing assays

The methods for transwell invasion assay have been described [25]. 24-well plates inserted with the membranes coated with Matrigel and prehydrated in serum-free medium were utilized. 2×10^4^ cells in complete medium without FBS were placed on the top chamber. Complete medium containing 10% FBS with or without PlGF-1 was added as a chemoattractant in the lower chamber. After 24 hours, cells on the upper side of the membrane were mechanically removed. The attached cells were counted under a light microscope. All assays were repeated at least three times independently.

Wound healing assays was done as described previously [14]. All samples were tested in triplicate.

### Cellular F-actin measurement

The F-actin content was done as described previously [26, 27]. The relative F-actin content was calculated by the following equations: F-actin Δt/F-actin 0=fluorescence Δt/fluorescence 0. All experiments were tested repeatedly for three times.

### Fluorescence microscopy

The cells were cultured 1 day before this experiment and then starved in serum-free medium for 3 hours. After the stimulation with 10 ng/ml PlGF-1 at 37°C for 1 min, cells were fixed with 4% paraformaldehyde and permeablized with 0.1% Triton X-100 in PBS. For staining actin, the cells were then directly incubated with Alexa-fluro 568 phalloidin (Cytoskeleton, Inc.) for 30 min in the dark and gently washed with PBS. The cells were then kept in the PBS and directly visualized with the Confocal microscope.

### Human breast cancer tissues

Breast tissue specimens were obtained from the Department of Pathology, Affiliated Hospital of Weifang Medical University from January 2012 to December 2014. These tissue specimens consisted of samples from 90 cases of paraffin-embedded specimens of invasive ductal carcinoma and 30 selected frozen (liquid nitrogen) invasive ductal carcinoma tissues. Prior donors' consents and approvals from Ethics Committee of the Weifang Medical University were obtained. Clinical information of the samples is described in detail in Table 1.

### *In vivo* assays for metastasis

For the experimental metastasis mouse xenograft model, MDA-MB-231 cells stably expressing miR-507 NC (MDA231/NC), miR-507 mimic lentiviral vector (MDA231/miR507), knocked-down Flt-1 expression (SiFlt-1/MDA231), the negative control (Scr/MDA231) were injected into their mammary fat pads of five-week-old female BALB/C-nu/nu nude mice (N = 10). The animals were purchased from Wei Tong Li Hua Experimental Animal Company. When the xenografts were palpable (~0.5 cm in diameter) in Scr/MDA231, SiFlt-1/MDA231 cell groups, an intratumor injection of rPlGF-1 at 10 ng/kg was performed biweekly for 4 consecutive weeks. The mice were manipulated and housed according to protocols by the Animal Care and Use Committee of the institution. After some weeks, the mice were sacrificed and the lung tissues were fixed with formalin and embedded in paraffin to examine metastasis. Serial sections and H&E staining were performed to detect lung micrometastasis.

### Bisulfite sequencing and DNA methylation

Genomic DNAs from MCF-10A, breast cancer cell lines (MCF-7 and MDA-MB-231), and clinical specimens were bisulphite modified with the Epitect Bisulphite Kit (Qiagen, Duesseldorf, Germany). Bisulphitetreated DNAs were amplified with BSP (bisulphite-sequencing PCR) primers located in the miR-507 promoter.

### 5-Aza-2′-deoxycytidine treatment

Breast cancer cells were seeded in 10cm dishes 24 hours before drug treatment. The cells were treated with 5 μM 5-aza-2′-deoxycytidine (5-aza-dC) every 24 hours for 3 days.

### Statistical analysis

All statistical analyses were carried out using the SPSS v16.0 software. Data are presented as mean ± SD. Statistical significance for comparisons between groups was determined using Student's paired two-tailed t-test or ANOVA. Chi-square test was used to analyze the relationship between miR-507 expression and the clinicopathologic characteristics. All the results were generated from three independent experiments. In all cases, *P* < 0.05 was considered statistically significant.
